# Treatable traits and challenges in the clinical management of non-tuberculous mycobacteria lung disease in people with cystic fibrosis

**DOI:** 10.1186/s12931-023-02612-1

**Published:** 2023-12-16

**Authors:** Andrea Gramegna, Sofia Misuraca, Andrea Lombardi, Chiara Premuda, Ivan Barone, Margherita Ori, Francesco Amati, Mariangela Retucci, Erica Nazzari, Gianfranco Alicandro, Maurizio Ferrarese, Luigi Codecasa, Alessandra Bandera, Stefano Aliberti, Valeria Daccò, Francesco Blasi

**Affiliations:** 1 Department of Pathophysiology and Transplantation, University of Milan, Via Francesco Sforza 35, 20122 Milan, Italy; 2 Respiratory Unit and Cystic Fibrosis Adult Center, Fondazione IRCCS Ca’ Granda Ospedale Maggiore Policlinico, Via Francesco Sforza 35, 20122 Milan, Italy; 3 Infectious Diseases Unit, Foundation IRCCS Ca’ Granda Ospedale Maggiore Policlinico, Milan, Italy; 4 Department of Biomedical Sciences, Humanitas University, Via Rita Levi Montalcini 4, Pieve Emanuele, 20072 Milan, Italy; 5 Respiratory Unit, IRCCS Humanitas Research Hospital, Via Manzoni 56, Rozzano, 20089 Milan, Italy; 6 Healthcare Professions Department, Foundation IRCCS Ca’ Granda Ospedale Maggiore Policlinico, Milan, Italy; 7 Cystic Fibrosis Center, Foundation IRCCS Ca’ Granda Ospedale Maggiore Policlinico, Via Commenda 9, 20122 Milan, Italy; 8 Regional TB Reference Centre, Villa Marelli Institute, Niguarda Hospital, Milan, Italy

**Keywords:** Cystic fibrosis, NTM pulmonary disease, Personalized medicine, Treatable traits

## Abstract

**Introduction:**

Over the last ten years an increasing prevalence and incidence of non-tuberculous mycobacteria (NTM) has been reported among patients with cystic fibrosis (CF) Viviani (J Cyst Fibros, 15(5):619–623, 2016). NTM pulmonary disease has been associated with negative clinical outcomes and often requires pharmacological treatment. Although specific guidelines help clinicians in the process of diagnosis and clinical management, the focus on the multidimensional assessment of concomitant problems is still scarce.

**Main body:**

This review aims to identify the treatable traits of NTM pulmonary disease in people with CF and discuss the importance of a multidisciplinary approach in order to detect and manage all the clinical and behavioral aspects of the disease. The multidisciplinary complexity of NTM pulmonary disease in CF requires careful management of respiratory and extra-respiratory, including control of comorbidities, drug interactions and behavioral factors as adherence to therapies.

**Conclusions:**

The treatable trait strategy can help to optimize clinical management through systematic assessment of all the aspects of the disease, providing a holistic treatment for such a multi-systemic and complex condition.

## Introduction

Cystic fibrosis (CF) is a lethal inherited disease characterized by multiorgan manifestations with morbidity and mortality primarily arising from CF-related lung involvement [1–3]. Systemic manifestations result from the impairment of the cystic fibrosis transmembrane conductance regulator (CFTR) protein, which regulates salt and water balance across epithelial cells. In the lungs, this dysfunction contributes to the loss of the airway surface liquid layer and a state of hyper-absorption, leading to an impairment of mucociliary clearance [4]. Pathogens, often non-fermenting gram-negative bacteria, may adapt to the host environment and cause chronic lung infection, thus leading to neutrophil-mediated chronic inflammation and irreversible bronchiectasis [5]. In this context, in recent years, an increasing prevalence and incidence of non-tuberculous mycobacteria (NTM) has been documented among patients with CF [1, 3, 6].

The prevalence of NTM infection in people with CF (pwCF) has varied dramatically over the last decades, with a recent metanalysis reporting a pooled estimate of 7.9% (95% CI 5.1–12.0%) [6–9]. The most common NTM species reported in pwCF are *Mycobacterium abscessus complex* (MABSC) and *Mycobacterium avium complex* (MAC) [1, 3, 6]. Clinical consequences of NTM infections in CF range from transient colonization to chronic infection. The latter can be indolent or contribute to radiographic changes and worsening of respiratory symptoms referred to as NTM pulmonary disease (NTM-PD), often requiring specific treatment [10]. NTM-PD has been associated with accelerated lung function decline and worse clinical outcomes in pwCF. Moreover, it poses a relative contraindication for lung transplant due to the NTM’s inherent resistance to antimicrobial therapy [11–14]. Given its increasing incidence and relevant clinical implications, NTM-PD specific guidelines have been developed for CF patients [10]. Floto and co-authors generated a series of pragmatic, evidence-based recommendations for screening, investigating, diagnosing, and treating NTM-PD infection in pw CF. The authors recommended that pwCF should be evaluated for NTM treatment if they meet the ATS/IDSA criteria for pulmonary disease and undergo drug susceptibility testing following the Clinical and Laboratory Standards Institute (CLSI) guidelines. In addition, the document also emphasized that CF pathogens and CF-related comorbidities should be considered potential confounders when NTM-PD is suspected.

This review aims to identify the treatable traits (TT) of NTM-PD in pwCF and discuss the importance of a multidisciplinary approach in order to detect and manage TTs with the aim of improving outcomes.

## Definition of treatable trait

A TT is a validated phenotypic or endotypic characteristic that can be assessed and successfully targeted by an intervention with the aim of improving a related clinical outcome [15]. The TT strategy was initially proposed for chronic obstructive pulmonary disease (COPD) and recently extended to asthma and interstitial lung disease patients[16, 17]. This strategy aims to classify treatable targets based on their nature and to treat patients following a precision medicine approach [18]. Agustì and co-authors divided potential TT of airways disease into three broad categories (pulmonary, extrapulmonary, and lifestyle-related TT) and discussed a possible hierarchy of traits in light of their clinical impact and available therapeutic options [18].

In such a scenario, pwCF and NTM-PD present a high complexity regarding comorbidities and concomitant medications. These aspects warrant careful consideration before initiating a specific NTM treatment and throughout the follow-up.

While the guidelines outlined the fundamental aspects of specific treatment for NTM-PD in pw CF, little space is devoted to the multidimensional assessment of concomitant problems [10]. The multidisciplinary complexity of NTM-PD in CF requires careful management of respiratory aspects, including airway clearance and treatment of chronic respiratory infection caused by CF pathogens (*respiratory traits*); control of comorbidities that may impact treatment outcomes, such in the case of CF-related diabetes and malnutrition (*extra-respiratory traits*), and behavioral factors as adherence to therapies (*lifestyle*). Finally, chronic and multidrug medications in CF pose challenges to NTM treatment initiation in terms of increased risk of resistance or drug-drug interactions (TT).

**Fig. 1:**
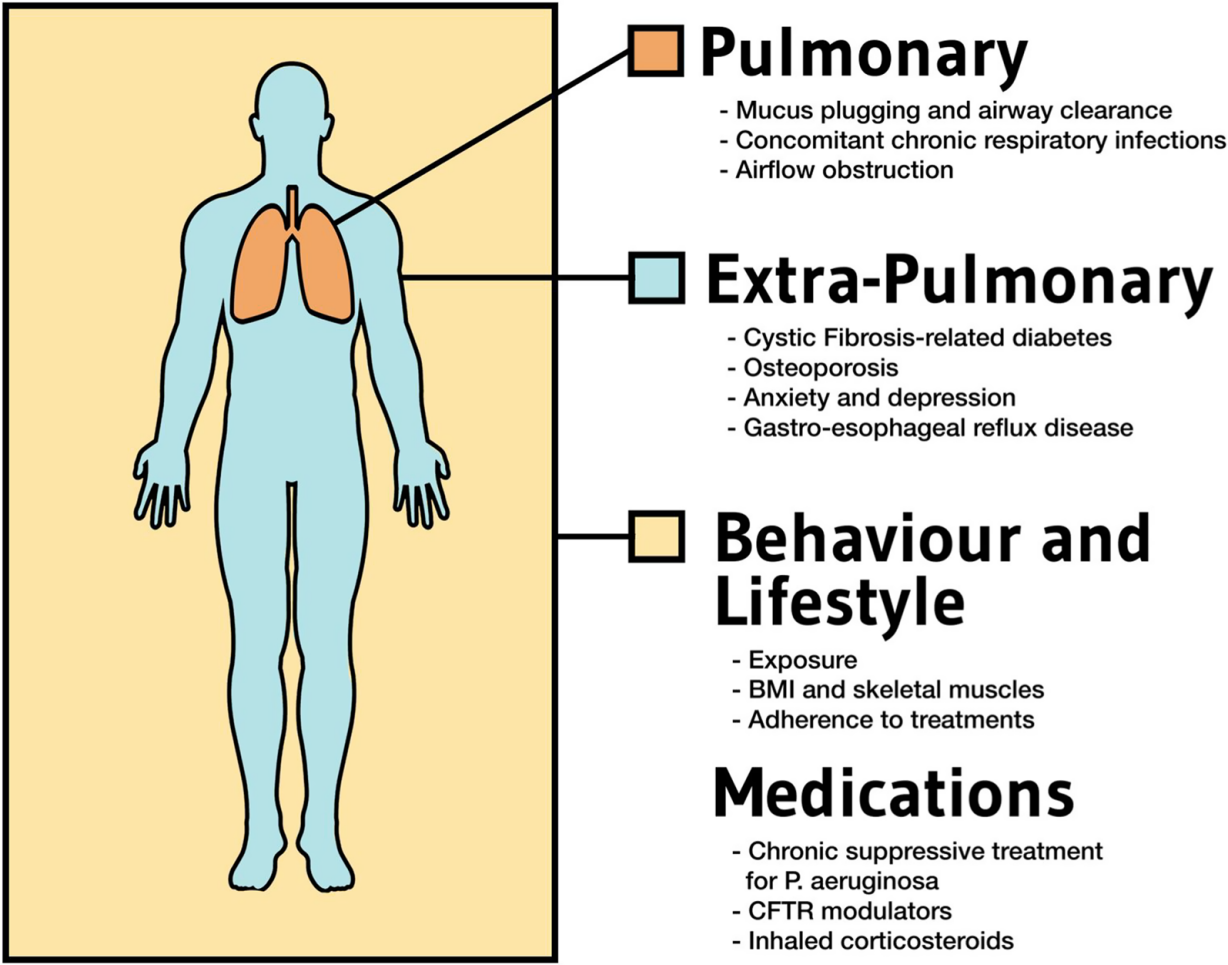
Overview of the treatable trait strategy to approach the clinical management of NTM pulmonary disease in people with cystic fibrosis

Figure 1 summarizes the different treatable traits in pwCF and NTM lung disease.

## Pulmonary traits

### Mucus plugging and airway clearance

The critical role of airway cleaning techniques (ACT) in improving lung function and preventing pulmonary exacerbations in pwCF is well established [19, 20]. Therefore, ACT is recommended for all pwCF and should be initiated at the time of diagnosis and performed daily [19, 21–23]. Given the absence of conclusive findings from observational studies or clinical trials favoring one technique over another, the choice of the optimal ACT should be tailored to individual patient’s needs and preferences [19, 24]. Considering the severity of the disease, the use of non-invasive ventilation might be considered [25]. In addition, ACT are often used in combination with aerosol osmotic agents (hypertonic saline and mannitol dry powder) or mucolytic agents (recombinant human DNAse) to improve rheological properties of bronchial secretions [26].

There is no evidence that ACT can reduce NTM infection or avoid progression to NTM-PD in pwCF. However, a study involving patients with bronchiectasis found a higher prevalence of peripheral mucus obstruction on CT scans in the NTM-positive group compared to the NTM-negative group, suggesting that increased mucus may create a favorable environment for NTM growth [27]. On the other hand, inadequate airway clearance may contribute to the worsening of respiratory symptoms as well as lung disease progression, which may add confusion when deciding to start a specific NTM treatment. Therefore, a multidisciplinary approach is important in order to promote and periodically reassess adherence to ACT in pwCF before and after the diagnosis of NTM-PD[28].

It is worth mentioning that in recent years the introduction of CFTR modulators has been changing the scenario [29]. This new class of drugs interferes with the folding of the mutant CFTR and partially corrects its biological function [30]. These treatments have been associated with a marked improvement in clinical outcomes secondary to their effect in improving airway clearance [31], hence the emerging need to reassess ACT in pwCF undergoing CFTR modulators therapy [32]. Nevertheless, the real impact of these novel treatments on NTM infection has yet to be determined. Registry data suggest a noteworthy association between the utilization of CFTR modulators and a significantly reduced prevalence of NTM culture positivity in pw CF [33].

### Concomitant chronic respiratory infections

Polymicrobial chronic respiratory infection is common in pwCF. As pwCF age and their disease progresses, the composition of respiratory pathogens in sputum evolves, with *P. aeruginosa* and other non-fermenting gram-negative bacteria being more prevalent among adults [34, 35]. Furthermore, a role in disease progression has also been increasingly recognized for fungi and viruses [34].

Symptoms and radiological characteristics of NTM-PD may exhibit similarities with those of other respiratory infections, making it hard to distinguish between NTM-PD and pulmonary disease caused by traditional CF pathogens in patients with positive cultures for both bacteria and NTM. For this reason, guidelines recommend using intravenous antibiotics for conventional bacteria in determining the clinical significance of NTM in sputum [10].

In recent years, many studies have focused on identifying the microorganisms most often associated with NTM infections and their possible impact on clinical outcomes. Discordant data of association or coexistence with NTM have been reported concerning bacteria. Some studies have shown higher rates of *P. aeruginosa* in NTM-positive sputum cultures, while others have found an association with *S. aureus* and *S. maltophilia* [10]. A recent meta-analysis supported this hypothesis, showing that *S. aureus* and *S. maltophilia-positive* cultures were associated with a higher risk of NTM positivity with an OR of 1.66 (95% CI: 1.21–2.26) and 3.41 (95% CI: 1.2–2.26), respectively [36].

Identification of fungal species in sputum samples is common in pwCF. It may be facilitated by many factors, such as impaired airway clearance, leading to increased exposure to inhaled fungal spores and frequent use of broad-spectrum antimicrobials, which could may favor fungal infection [37]. Co-existence between NTM and fungi is documented in pwCF and bronchiectasis patients, particularly concerning *Aspergillus fumigatus* [10, 38]. The meta-analysis mentioned above also reported an increased risk for NTM-positive cultures (OR 3.59; 95% CI: 3.05–4.23) in patients with *A. fumigatus* colonization [36].

In people with NTM-PD, co-infection between NTM and *A. fumigatus* was associated with higher mortality than NTM alone [39]. The clinical management of these conditions can be challenging because it is difficult to discriminate which pathogen impacts the most on clinical outcomes and because of drug interactions between oral triazoles and antimycobacterial agents [39].

Allergic bronchopulmonary aspergillosis (ABPA) has also been indicated as a risk factor for NTM infection [10]. One of the reasons for this association could be that *A. fumigatus* induces a Th2-mediated immune response, thus reducing the cytokines involved in NTM eradication [40]. Another possible explanation could be represented by the prolonged exposure to systemic and inhaled steroids, a known risk factor for NTM-PD [40].

### Airflow obstruction

Progressive airway obstruction in pwCF can arise from reversible and irreversible causes, such as mucus obstruction and bronchial inflammation on the one hand and airway remodeling and progressive bronchial damage on the other [41, 42]. Although the evidence regarding the use of bronchodilators is inconclusive and guidelines provide conflicting recommendations, chronic prescription of long-acting inhaled bronchodilators is a common strategy to control airway obstruction and prevent lung function decline in pwCF-related lung disease [43, 44].

Airway obstruction has also been documented in NTM-PD, and a low percent predicted Forced Expiratory Volume in the first second FEV1 (ppFEV1) was associated with an increased risk of unfavorable outcomes in this population [13, 45]. A trend towards a decline in lung function was reported for NTM culture-positive patients during the year before the first isolation, with an acceleration in the case of NTM-PD [46]. In addition, NTM-PD was associated with a lower baseline ppFEV1 and a faster decline in lung function over time in comparison with patients with NTM isolation [46, 47]. Optimizing the treatment of airway obstruction using single or double bronchodilation is a reasonable approach that should be recommended early in the diagnostic evaluation and treatment of NTM-PD.

## Extrapulmonary traits

### CF-related diabetes (CFRD)

CFRD is a common extrapulmonary complication of CF affecting 50% of patients over the age of 30, with a prevalence increasing with age [48]. CFRD is one of the most important determinants of disease progression, as it is associated with a faster decline in pulmonary function and increased mortality [49].

In a recent large study exploring the association between diabetes and NTM-PD, Wang and co-authors demonstrated that people with diabetes have an increased risk of developing NTM-PD [50]. One possible explanation comes from the impact that diabetes may have on immune response, making patients more susceptible to NTM infections. Furthermore, several studies have also investigated the association between diabetes and tuberculosis. Some authors found that individuals with poorly controlled diabetes, as indicated by elevated fasting glucose or hemoglobin A1c levels, are more susceptible to developing active tuberculosis [51–53]. Supporting the validity of this hypothesis also for NTM-PD in pwCF, it is worth noting that CFRD rates are higher in patients with NTM infection than in patients without NTM, with other markers of clinical severity such as malnutrition and underweight [11]. There is currently limited evidence on NTM-PD as an independent risk factor for CFRD [54].

Finally, it is worth mentioning as a concern for clinicians taking care of patients with CFRD and NTM infection that some of the antimicrobials used in NTM-PD treatment may have potential interactions with anti-diabetes drugs, as in the case of clarithromycin and sulfonylureas.

### Osteoporosis

PwCF are at increased risk of developing osteoporosis due to several factors, including malabsorption of vitamins and minerals, hormonal dysfunction, undernutrition, and reduced physical activity often seen in severe disease[55] [56]. Osteoporosis and NTM-PD share a number of risk factors, which may contribute to their co-existence in this population. Furthermore, NTM-PD has been associated with a distinctive body phenotype characterized by lower body mass index (BMI) and altered serum adipokine levels. At the same time, the role of bone metabolism has not yet been defined [57]. Female sex is also a risk factor for NTM-PD and osteoporosis, especially among middle-aged and elderly individuals [58, 59].

In light of all these factors, the coexistence of NTM-PD and osteoporosis appears frequent, as reported in some cohorts [60, 61]. Thus osteopenia/osteoporosis in NTM-PD patients represent a relevant TT that deserves specific evaluation and treatment [58]. Further studies should consider better endophenotypic profiling of osteoporosis patients and investigate the impact of bone disease and its treatment on clinical outcomes of NTM-PD.

### Anxiety and depression

Several studies have highlighted the association between CF and mental conditions, such as anxiety and depression (AD) [62]. AD in CF is known to affect prognosis, medication adherence, and overall quality of life [63–65]. The role of AD in NTM-PD has been studied less extensively, although recent studies have investigated the incidence of AD in this population [66, 67]. There are currently no studies specifically investigating AD in CF and NTM-PD patients. It can be assumed that these patients may experience mental problems due to prolonged and multidrug treatments, drug side effects, and uncertain clinical outcomes.

Mental health problems in pwCF and NTM-PD can have a major impact on medication adherence and patient report outcomes. Therefore, adequate screening and psychological support during NTM treatment is desirable. Designing and conducting studies that address this issue should require careful consideration of multiple factors, such as the stage of NTM infection, treatment protocols, and individual differences in coping mechanisms.

### Gastroesophageal reflux disease (GERD)

PwCF have many risk factors for GERD, including chronic cough, over-eating to counter intestinal malabsorption, delayed gastric emptying, and frequent position changes related to ACT [68, 69]. As a consequence, the prevalence of GERD in pwCF is high, ranging from 35 to 81% in different observational cohorts with a relevant heterogeneity related to the different definitions [70, 71].

A large retrospective population-based cohort study by Kim and co-authors demonstrated that GERD is associated with an increased risk of developing NTM-PD (HR 3.36; 95% CI, 2.10–5.37) and that older age and bronchiectasis are risk factors for NTM infection in patients with GERD [72]. Possible explanations are the pro-inflammatory action of acid reflux into the airways and the potential role of the gastrointestinal tract as a reservoir for microbes relevant to CF lung pathophysiology [73, 74]. NTM are no exception, as shown by Dawrs and co-authors in a recent in vitro study, where it was hypothesized that NTM could survive and cause infection in bronchial cells during episodes of gastroesophageal reflux and microaspiration after being ingested from drinking water or other environmental sources [75].

The increased prevalence of GERD in pwCF may act as an additional risk factor for NTM-PD, and the multidisciplinary team must be aware of this concurrent condition and provide simultaneous treatment.

## Behavior and lifestyle

### Avoiding exposure to NTM

It is thought that NTM is primarily acquired from environmental sites, including soil and water, as well as from water supply systems to homes, hospitals, and clinics. Aerosols generated by flowing water from taps, showers and fountains are also potential sources [76]. Living in an urban versus a rural setting has been associated with different epidemiology of NTM infection, e.g., living in an area of higher population density is associated with *M. kansasii* infection, whereas rural areas are associated with MAC [77]. Studies of the homes of NTM-PD patients have found NTM isolates in showerheads, bathtub water, drain outlets, humidifiers, heating/ventilation systems, bathroom inlets, bathroom and kitchen faucets, and refrigerator taps [76, 78].

While inhalation of droplets is the most common route of respiratory infection, nosocomial infection has often been hypothesized, especially in pwCF, where cross-infections from bacterial pathogens is well documented. In the last decade, a series of hospital-associated outbreaks of MABSC within CF centers have been suspected [79–81]. Aitken and colleagues reported five patients who had overlapping clinical encounters at the center and were found to have highly similar isolates of *M. abscessus subspecies massiliense.* They speculated that the index case may have contaminated the clinic environment facilitating indirect patient-to-patient transmission [80]. A similar study in Hawaii reported an outbreak of *M. abscessus subspecies Abscessus* in which 9 of 19 pwCF were identified with identical NTM isolates. The authors concluded that using shared pulmonary function testing tools likely led to MABSC contamination of the laboratory from an index case and the subsequent spread of NTM to other patients [81]. On the other side, Tortoli and colleagues identified very few highly clustered cases in pwCF. Specifically, the authors investigated the whole genome level of MABSC isolated from all patients attending four Italian CF centers [82].

The results of these studies suggest that the role of cross-infection for NTM is still debated, even if Bryant and colleagues reported outbreaks in pwCF as consequence to direct or fomite-mediated transmission[83]. Therefore, it is advisable to continue practicing segregation of patients at CF centers.

### Improving BMI

Despite early nutritional management and pancreatic enzyme replacement therapy, underweight remains an essential concern in pwCF at all ages, especially in countries with suboptimal standards of care. Being underweight in CF is caused by malabsorption of nutrients and increased energy expenditure sustained by chronic inflammation and increased respiratory efforts [84–87].

Underweight is considered a negative prognostic factor in NTM-PD [88]. In a retrospective cohort study including 663 patients diagnosed with NTM-PD, Sung Woo and co-authors found that underweight was a significant risk factor for all-cause mortality. Moreover, they observed that treatment intolerance was related to malnutrition as defined by the prognostic nutritional index at the time of treatment initiation [89].

Campbell and colleagues conducted a meta-analysis to explore the role of BMI at initiation of rifampicin-resistant tuberculosis treatment. Authors concluded that low BMI at the beginning of therapy is associated with increased odds of unfavorable treatment outcomes, particularly mortality [90].

While these findings underscore the importance of adequate nutritional status in patients with NTM-PD, there is a paucity of studies examining nutritional endpoints, such as dietary intake, long-term energy balance, and skeletal muscle mass, in this population [91].

### Promoting adherence to treatments

Chronic treatment to maintain good health requires considerable time and effort for pwCF. This leads to ongoing challenges to patient self-management strategies, particularly in adults trying to balance family, work, and education [92]. Not surprisingly, therefore, studies on this topic suggest that levels of adherence to treatments are very low, although they vary according to the type of treatment [93]. Suboptimal adherence can negatively impact health outcomes such as the rate of pulmonary exacerbations, quality of life, and health care costs [94–97]. For this reason, several interventions such as telemonitoring, improved accessibility to the CF center, and psychological interventions [98, 99].

In NTM-PD, adherence becomes even more relevant, as treatment is burdensome, complicated, and usually takes 18–24 months [100, 101]. Due to adverse events and drug-drug interactions, many patients are at risk of premature termination of treatment [102].

Promoting adherence is, therefore, one of the most significant TT in the clinical management of patients with NTM-PD. One of the possible strategies is the so-called ‘directly observed therapy’ (DOT), which involves a trained healthcare worker providing the prescribed drugs and watching the patient swallow each dose. DOT has been the standard for TB treatment for thirty years, especially for patients with drug-resistant TB [103]. Another strategy is simplifying NTM treatment by reducing it to 3 times a week (TIW). Multidrug TIW with macrolide, rifampicin, and ethambutol is a reasonable initial treatment regimen for patients with nodular/bronchiectatic MAC lung disease. At the same time, its use is not recommended for cavitary disease or other NTM species [10, 104].

Last, great attention is given to the concept of the therapeutic alliance, which means that the patient should be a key player in the choice to start therapies, aware of the possible side effects and length of treatment. Forming a partnership between physicians and patients allows for considering the patient's ability to accept the disease and sharing treatment goals [105].

## Challenges with concomitant medications

### Long-term macrolide treatment

Treatment with low-dose azithromycin is beneficial in pwCF and chronic *P. aeruginosa* mainly due to its immunomodulatory and anti-inflammatory properties [106]. The efficacy of macrolides does not appear to derive from the antibiotic effect but is mediated by inhibiting biofilm formation and other bacterial virulence factors. This results in improved lung function and reduced exacerbation rates in this group of patients [107].

Macrolides are also a key drug in the clinical management of NTM-PD. Current recommendations suggest azithromycin as a first-line drug in treating both MAC and MABSC-PD in CF [10].

Recently, attention has been paid to the impact of long-term macrolide monotherapy as a risk factor for both NTM acquisition and the emergence of macrolide-resistant strains. Azithromycin blockade of the autophagic killing of NTM within macrophages may be one of the possible mechanisms under this process [108]. However, while the association between NTM and azithromycin use has been repeatedly refuted, concerns are high about the potential development of macrolide-resistant NTM strains [47, 106, 109].

### Chronic suppressive treatment for *P. aeruginosa*

Using inhaled aminoglycosides is a common strategy in managing CF lung disease when chronic *P. aeruginosa* is present [110]. Inhaled tobramycin has been shown to reduce airway bacterial density, decrease the frequency of exacerbations, and improve quality of life and lung function in patients with chronic *P. aeruginosa* infection [111].

At the same time, aminoglycosides (especially amikacin) are recommended for the treatment of cavitary MAC-PD and MABSC-PD. It has been hypothesized that long-term exposure to inhaled tobramycin may increase the occurrence of amikacin-resistant NTM in pwCF.

Co-infection with *P. aeruginosa* and NTM poses significant challenges to the clinical management of pwCF, and clinicians should carefully consider the risk that chronic exposure to inhaled tobramycin may limit therapeutic options in a possible future treatment for NTM-PD.

### CFTR modulators

The recent advent of CFTR modulators, a new class of drugs that induce post-translational modifications in the CFTR mutated protein, has demonstrated a transformative impact on airway clearance and lung function in pwCF. Although unable to have a direct antimicrobial effect, the improvement in the microbiological niche of CF bronchiectasis has been associated with a reduced risk of new pulmonary infections [91]. A recent study by Ricotta and colleagues reported that the risk of having an NTM-positive culture decreased by 14% in individuals who received CFTR modulators compared to controls [33].

A residual concern emerges for those patients who receive a diagnosis of NTM-PD while on CFTR modulators. All components of the currently approved CFTR modulator therapy (elexacaftor, tezacaftor, lumacaftor, and ivacaftor) have shown hepatic metabolism primarily via cytochrome P450 3A [112] and may have potential drug-drug interactions.

The use of rifamycins, both rifabutin, and rifampicin, may encounter problems with concurrent use of ETI. A recent pharmacokinetic study demonstrated that rifampicin administration reduced ivacaftor concentration time by 89% [113]. This drug-drug interaction can potentially compromise the efficacy and safety of NTM treatment in this subgroup. The pharmacokinetic profile of rifabutin appears to be more moderate than that of rifampicin and suggests that its use with an adjusted dosing regimen could offer a pivotal alternative to rifampicin in patients receiving CFTR modulators.

Similar concerns are also present for clofazimine and clarithromycin. Hong and colleagues evaluated the co-administration of rifabutin, clofazimine, and clarithromycin using a multiparameter model and demonstrated significant drug interactions with elexacaftor, requiring dose adjustment [113]. The authors also found that the altered elexacaftor concentrations continued after discontinuing the NTM treatments and that resumption of the standard dose of ETI had to be delayed.

Further studies will help tailor the optimal dosage of NTM antimicrobials in consideration of drug-drug interactions for patients with limited treatment options.

### Inhaled steroids (ICS)

Although often overprescribed, the role of ICS remains largely unproven in the long-term management of pwCF unless in the presence of atopic asthma or bronchial hyper-responsiveness [114, 115].

Recent studies suggest that the use of ICS is associated with an increased risk of NTM-PD by tapering down cellular immunity against intracellular pathogens [116–119]. It is, therefore, reasonable to limit the use of chronic ICS in individuals with concomitant asthma and to consider ICS withdrawal when not strictly indicated.

## Conclusion

The clinical management of pwCF requires a high level of care provided by a multidisciplinary team. Managing patients becomes even more challenging in the case of concurrent NTM-PD, given the complexity of antimicrobial regimens and the potential impact on quality of life. The TT strategy can pave the way for optimizing clinical effort through systematic assessment of all clinical and behavioral aspects of the disease, providing a holistic treatment for such a multi-systemic and complex condition.

Table 1 shows our proposal for the TT of NTM-PD in this population and their clinical management.

**Table 1:** Proposed treatable traits of NTM lung disease in people with cystic fibrosis and their clinical management

Treatable Traits	Clinical figures	Clinical management
Mucus plugging	Respiratory physiotherapist	Airway clearance techniques; mucoactive adjuncts; pulmonary rehabilitation
CF pathogen chronic respiratory infection	CF specialist Infectious disease specialist	Systemic antibiotics (if acute phase); inhaled antibiotics (for either eradication of new pathogens or chronic suppression)
Airflow obstruction	CF specialist Pulmonologist	Long-acting inhaled bronchodilators (both LABA and LAMA); ICS (if hyper-responsiveness demonstrated)
CF-related diabetes	CF specialist Diabetologist	Referral to diabetology service
Osteoporosis	CF specialist Endocrinologist	Vitamin D/cholecalciferol supplementation; referral to bone health service
Anxiety and depression	Psychiatrist Psychologist	Psychological support; referral to the mental health service
GERD	CF Dietician Gastroenterologist	Dietary restrictions; proton pump inhibitors; antacids; prokinetics; referral to the gastroenterology service
Environmental exposure	CF specialist Respiratory physiotherapist	Avoid risky activities (fishing, gardening, hot springs); segregation at CF center; disinfection of devices
Undernutrition and performance status	CF dietician	Nutritional screening at each clinical encounter; assessment of energy and nutrient requirements; individual dietary counseling to maintain optimal nutritional status and avoid undernutrition and excessive weight gain; Physical training
Adherence to treatments	CF specialist	Tele-monitoring; easy access to CF center; psychological support; directly observed therapy; three times weekly therapy
Risk of NTM-DR during long-term azithromycin	CF specialist Pulmonologist Infectious disease specialist	Rule out NTM before starting azithromycin; evaluate azithromycin discontinuation in case of NTM occurrence; optimize ACT and chronic treatment
Risk of NTM-DR during inhaled aminoglycosides	CF specialist Pulmonologist Infectious disease specialist	Rule out NTM before starting inhaled treatment; evaluate aminoglycoside discontinuation in case of NTM occurrence (shift to other inhaled antibiotics); optimize ACT and chronic treatment
Interactions with CFTR modulators	CF specialist Pulmonologist Infectious disease specialist	Evaluate rifabutin instead of rifampicin; check drugs interactions; CFTR modulator dose adjustment
Avoiding inhaled corticosteroids	CF specialist Respiratory physiotherapist	Test for bronchial hyper-responsiveness; evaluate safe ICS withdrawal

*CF* cystic fibrosis, *LABA* long acting beta-2 agonists, *LAMA* long acting anti-muscarinic agents, *ICS* inhaled corticosteroids, *GERD* gastro-esophageal reflux disease, *NTM-DR* drug resistant non-tuberculous mycobacteria, *ACT* Airway clearance technique

Finally, the advent of CFTR modulators has increased life expectancy in pwCF to a median survival of 60 years or more in high-income countries [120]. This brings new challenges, including treating CF in an aging population with age-dependent comorbidities. These aspects should be carefully considered in the global assessment of the patient with NTM-PD and CF.

## Data Availability

Not applicable.

## Abbreviations

CF: Cystic fibrosis
CFTR: Cystic fibrosis transmembrane conductance regulator
NTM: Non-tuberculous mycobacteria
pwCF: People with CF
MABSC: *Mycobacterium abscessus complex*
MAC: *Mycobacterium avium complex*
NTM-PD: NTM pulmonary disease
CLSI: Clinical and Laboratory Standards Institute
TT: Treatable traits
COPD: Chronic obstructive pulmonary disease
ACT: Airway cleaning techniques
ppFEV1: Percent predicted Forced Expiratory Volume in the first second FEV1
CFRD: CF-related Diabetes
AD: Anxiety and depression
GERD: Gastroesophageal reflux disease
